# Human papillomavirus prevalence in male and female university students in Gaborone, Botswana

**DOI:** 10.1017/S0950268822000619

**Published:** 2022-04-06

**Authors:** Doreen Ramogola-Masire, Nancy McClung, Anikie Mathoma, Julia W. Gargano, Naledi Gape Nyepetsi, Troy D. Querec, Juanita Onyekwuluje, Madisa Mine, Chelsea Morroni, Rebecca Luckett, Lauri E. Markowitz

**Affiliations:** 1 Faculty of Medicine, Department of Obstetrics and Gynecology, University of Botswana, Gaborone, Botswana; 2 National Center for Immunization and Respiratory Diseases, Centers for Disease Control and Prevention, Atlanta, Georgia, USA; 3 Faculty of Medicine, Office of Research and Graduate Studies, University of Botswana, Gaborone, Botswana; 4 Botswana-University of Maryland School of Medicine, BUMMHI, Gaborone, Botswana; 5 National Center for Emerging, Zoonotic, and Infectious Diseases, Centers for Disease Control and Prevention, Atlanta, Georgia, USA; 6 National Health Laboratory, Botswana Ministry of Health and Wellness, Gaborone, Botswana; 7 Botswana Harvard AIDS Institute Partnership, Gaborone, Botswana; 8 MRC Centre for Reproductive Health, University of Edinburgh, Edinburgh, Scotland; 9 Beth Israel Deaconess Medical Center, Department of Obstetrics and Gynecology, Boston, MA, USA

**Keywords:** Botswana, females, HPV vaccine, human papillomavirus, males

## Abstract

In 2015, Botswana introduced the quadrivalent human papillomavirus (HPV) vaccine as a two-dose schedule in girls aged 9–13 years. We sought to establish a baseline HPV prevalence in unvaccinated young adults in Botswana. HIV-uninfected men and women aged 18–22 years were recruited from the University of Botswana in Gaborone during October 2019–February 2021. Demographic and behavioural characteristics were self-reported during structured interviews. Self-collected vaginal and penile swabs were tested for 28 HPV types using Seegene Anyplex II HPV28. We compared any HPV type, quadrivalent vaccine (HPV 6, 11, 16, 18)-type and non-quadrivalent vaccine-type prevalence in men and women and evaluated the risk factors for prevalence of any HPV type. A total of 493 men and 500 women were included in the analysis. Compared to men, women had higher prevalence of any HPV type (63.0% *versus* 31.4%, *P* < 0.001), vaccine-type HPV (21% *versus* 9.7%, *P* < 0.001) and non-vaccine-type HPV (60.4% *versus* 28.4%, *P* < 0.001). Higher prevalence of any HPV type in men and women was associated with having ≥2 sex partners in the past 12 months; always using condoms in the past 3 months was associated with a lower HPV prevalence. These data provide baseline information for future evaluation of the population impact of the HPV vaccination programme, including potential herd effects in men.

## Introduction

Human papillomavirus (HPV) infection causes cervical cancer as well as other anogenital cancers (anal, vulvar, vaginal and penile) and oropharyngeal cancers [1]. HPV types 16 and 18 are associated with about 70% of cervical cancer cases worldwide [2]. Worldwide, there is a substantial burden of cervical cancer with approximately 600 000 cervical cancer cases, and 300 000 deaths per year [3]. Cervical cancer is the most common cancer in women in Botswana and elsewhere in sub-Saharan Africa [3].

Highly effective prophylactic HPV vaccines have been developed and were introduced in many high-income countries starting in 2006 [4]. All three available HPV vaccines, the bivalent vaccine, the quadrivalent vaccine and the 9-valent vaccine, target HPV 16 and 18. The quadrivalent and 9-valent vaccines also target HPV 6 and 11, which cause most anogenital warts; the 9-valent vaccine also targets 5 additional high-risk (HR), i.e. cancer-causing HPV types [5, 6]. While all HPV vaccines were originally studied and licensed as a 3-dose schedule, in 2014 the World Health Organization (WHO) recommended a 2-dose schedule for persons starting the series before age 15 years [7]. A 3-dose schedule is recommended for those starting vaccination on or after the 15^th^ birthday, and for persons with immunocompromising conditions, including human immunodeficiency virus (HIV) infection.

Botswana's first comprehensive national cervical cancer control strategy focused on secondary prevention through cervical cancer screening. In 2015, after conducting an HPV vaccine demonstration project, Botswana introduced HPV vaccine into their national immunisation programme [8, 9]. National vaccine introduction in Botswana started in early 2015, using the quadrivalent HPV vaccine as a 2-dose schedule. The vaccine was delivered through school-based programmes in a 0, 6–12-month schedule. During the first year of implementation, girls aged 9–13 years were vaccinated, followed by 9-year-old girls in subsequent years. Over 90% 2-dose coverage has been achieved in the target age groups. Botswana was the second country in Africa to introduce HPV vaccination into their national programme and the first country in Africa to introduce a 2-dose vaccination schedule [10].

**Fig. 1.**
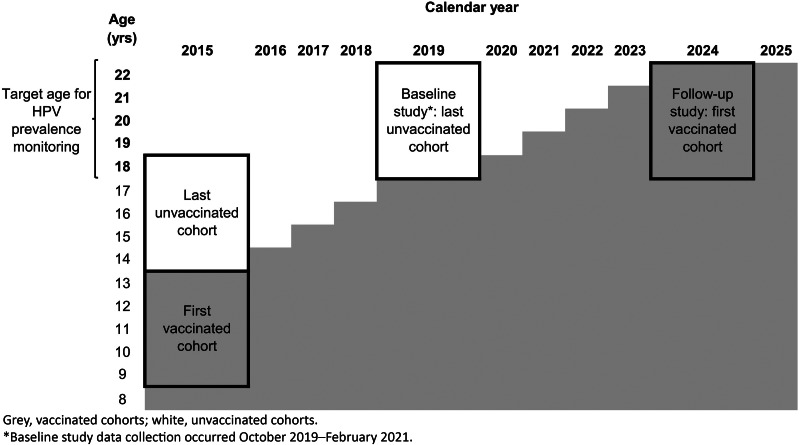
Description of calendar years and age groups for evaluation of a baseline HPV prevalence and future HPV vaccine impact in Botswana.

Measuring the impact of HPV vaccination after introduction into national programmes has focused on early and intermediate outcomes of HPV infection, as HPV-associated cancers take years to decades to develop. Reduction in the prevalence of HPV vaccine-targeted types has been observed soon after introduction (within 2–5 years); data through 10 years after the start of programmes are available from some high-income countries [11, 12]. Further, herd protection has been observed in unvaccinated young women and men in countries with female-only vaccination [11, 12]. HPV vaccine impact studies have not yet been conducted in Botswana. Although the HPV vaccination programme began in 2015, a baseline HPV prevalence could still be established in women aged 18–22 years because girls vaccinated in the first year of the programme were aged 13–17 years in 2019 (Fig. 1). Although there is no vaccination programme for boys in Botswana, a baseline HPV prevalence in young adult men will allow for an evaluation of herd protection. Thus, the aims of this study were to (1) evaluate HPV prevalence in young adults aged 18–22 years in Botswana, in females as a baseline for future vaccine impact monitoring, and in males as a baseline for future evaluation of herd protection from the female-only vaccination programme and (2) determine risk factors for HPV prevalence in females and males.

## Materials and methods

### Study design and procedures

We recruited a convenience sample of male and female students aged 18–22 years from the University of Botswana, in Gaborone, Botswana from October 2019 to February 2021. A comparison of HPV prevalence among women living with HIV and women without HIV from this study was published previously [13]. Participants were recruited directly from three main student congregation centres, and indirectly through fliers and notice board announcements. Study visits were initiated by students visiting the study site; although refusals after learning more about the study were not documented systematically, refusal occurred infrequently (i.e., <5%). All participants were required to either provide sufficient documentation of a negative HIV test within 12 months as recommended by Botswana HIV Care Clinical guidelines or participate in HIV counselling and testing at the study site by trained research assistants. Females who reported being pregnant were excluded from the study. Informed consent was obtained from all the participants prior to completing a short questionnaire of demographic and behavioural questions administered by research assistants. After the survey, research assistants provided illustrated instructions and explained how to self-collect specimens (vaginal or penile swab including glans, all sides of shaft and underneath the foreskin in uncircumcised participants). The study used a flocked swab (Copan Diagnostics, Murrieta, California, USA) which the participant placed in Specimen Transport Medium (STM) (Digene STM, Qiagen LLC, Germantown, Maryland, USA) after collection. Research assistants informed participants of any HR-HPV detection. The study was approved by the institutional review boards of the Centers for Disease Control and Prevention, the University of Botswana, and the Botswana Ministry of Health and Wellness.

### HPV testing

DNA was isolated from specimens with proteinase K lysis followed by automated extraction. STM swab specimens were agitated on an orbital shaker for 45 min at ambient room temperature. For specimen lysis, 400 μl of penile swab STM or 200 μl of vaginal swab STM was incubated with 20 μl proteinase K (catalog number CMG-1077, Perkin Elmer, Shelton, CT, USA) and enough NucliSENS easyMAG Lysis Buffer (catalog # 280134, bioMérieux SA, Marcy-l'Étoile, France) to bring the total processing volume to 800 μl. After incubation at 65 °C for 1 h, extraction was completed on NucliSENS EasyMAG (bioMérieux). DNA was eluted in 100 μl of buffer 3 at a pH >8.0 and a temperature of 70 °C. Sterile water aliquots were processed in parallel with specimens as negative controls for DNA contamination assessment.

Extracts were tested according to manufacturer End Point-CMTA protocol with Anyplex II HPV28 assay (catalog # HP7S00X; Seegene, Seoul, Korea). This assay detects 28 HPV types (6, 11, 16, 18, 26, 31, 33, 35, 39, 40, 42, 43, 44, 45, 51, 52, 53, 54, 56, 58, 59, 61, 66, 68, 69, 70, 73 and 82) and human internal control DNA. The assay was conducted on CFX96 Dx System (catalog #IBRD1855196, Bio-Rad, Hercules, California, USA). HPV typing results were considered invalid if both internal controls were negative and no HPV was detected.

### Statistical analysis

This analysis was limited to participants without evidence of HIV infection, who had valid HPV typing results. All analyses were performed using SAS 9.4 (Statistical Analysis Software, Cary, NC, USA). Demographic variables included age, sex and marital status (single, married, living together). Sexual and behavioural characteristics included being sexually experienced (defined as reporting any oral, anal, or vaginal sex), lifetime number of sex partners (0, 1–2, 3–4, 5–10, >10), number of sex partners in the past 12 months (1, 2, ≥3), age at first sex (<18, ≥18 years), age of first sex partner (<20, ≥20 years), age of current sex partner (<22, ≥22 years), sexual orientation (heterosexual/straight, homosexual/gay or lesbian, bisexual), condom use in past 3 months (always, sometimes/never, no sex in past 3 months), tobacco use (never, daily, weekly); in females, ever pregnant (yes/no) and in males, circumcised (yes/no) and age of circumcision (0–10, 11–15, 16–22 years).

For individual HPV types, all detected types were reported. HPV types were also categorised as any HPV, quadrivalent vaccine-types (HPV 6, 11, 16, 18), HR vaccine types (HPV 16, 18), low-risk (LR) vaccine types (HPV 6, 11), non-vaccine types (HPV 26, 31, 33, 35, 39, 40, 42, 43, 44, 45, 51, 52, 53, 54, 56, 58, 59, 61, 66, 68, 69, 70, 73, 82), non-vaccine HR types (HPV 31, 33, 35, 39, 45, 51, 52, 56, 58, 59, 66, 68) and non-vaccine LR types (HPV 26, 40, 42, 43, 44, 53, 54, 61, 69, 70, 73, 82). Type categories are not mutually exclusive, as a participant could have both HR and LR types, and both vaccine types and non-vaccine types, detected.

Demographic and behavioural characteristics were described overall and by sex. HPV prevalence was also described by sex, in all participants for both individual HPV types and HPV type categories, and in sexually experienced participants for HPV type categories. Any HPV, vaccine, and non-vaccine-type HPV prevalence by sex was further described by selected demographic and behavioural characteristics. Chi-square tests were used to identify statistically significant differences in categorical variables (*P* < 0.05). Bivariate analyses were conducted to determine participant characteristics associated with any HPV prevalence. In sexually experienced females and males, unadjusted prevalence ratios (PR) and 95% confidence intervals (CI) were calculated using log-binomial regression to evaluate associations between participant characteristics and any HPV type. Multivariable models were developed using backward selection (*P* < 0.3 to enter, *P* < 0.15 to stay) to identify characteristics independently associated with having a prevalent HPV infection in this population. Unadjusted and adjusted prevalence ratios (aPR) and 95% CI are reported for all variables included in the final model.

## Results

Of 500 HIV seronegative female and 500 HIV seronegative male participants, 500 (100%) females and 493 (98.6%) males had valid HPV results and were included in the analysis. Females and males had a similar distribution of ages and nearly all participants were single (99.8%) (Table 1). A similar proportion of females (74.4%) and males (76.3%) reported being sexually experienced. Males, however, were more likely than females to report ≥6 lifetime sex partners (20.3% *versus* 7.2%, *P* < 0.001). Males were more likely than females to report first sex at age <18 years (42.6% *versus* 17.5%, *P* < 0.001), younger ages at first sex and current sex partners (*P* < 0.001), and always using condoms in past 3 months (50.3% *versus* 44.9%, *P* = 0.02). In females, 8.4% reported ever being pregnant. In males, 60.9% reported being circumcised; of these, 84.0% reported circumcision prior to age 16 years.

**Table 1.** Demographic and behavioural characteristics of study participants by sex

	Females *N* = 500 *n* (%)	Males *N* = 493 *n* (%)	*P*-value†
Age (years)			
18	83 (16.6)	60 (12.2)	0.09
19	182 (36.4)	165 (33.5)	
20	97 (19.4)	120 (24.3)	
21	91 (18.2)	105 (21.3)	
22	47 (9.4)	43 (8.7)	
Marital status			
Married	0 (0)	0 (0)	–
Single	499 (99.8)	492 (99.8)	
Living together	1 (0.2)	1 (0.2)	
Sexually experienced			
Yes	372 (74.4)	376 (76.3)	0.49
No	128 (25.6)	117 (23.7)	
No. sex partners, past 12 months*			
0–1	231 (62.1)	156 (41.5)	<0.001
≥2	141 (37.9)	220 (58.5)	
Lifetime no. sex partners*			
0	128 (25.6)	117 (23.7)	<0.001
1	128 (25.6)	63 (12.8)	
2	105 (21.0)	64 (13.0)	
3–5	103 (20.6)	149 (30.2)	
≥6	36 (7.2)	100 (20.3)	
Age at first sex (years)*			
≥18	307 (82.5)	216 (57.5)	<0.001
<18	65 (17.5)	160 (42.6)	
Age of first sex partner (years)*			
≥20	235 (63.3)	63 (16.8)	<0.001
<20	136 (36.7)	313 (83.2)	
Age of current sex partner (years)*			
≥22	205 (55.3)	27 (7.2)	<0.001
<22	166 (44.7)	348 (92.8)	
Sex identity			
Heterosexual/straight	482 (96.4)	483 (98.0)	0.004
Homosexual/gay or lesbian	1 (0.2)	6 (1.2)	
Bisexual	16 (3.2)	4 (0.8)	
Other	1 (0.2)	0 (0)	
Condom use past 3 months*			
Always	167 (44.9)	189 (50.3)	0.02
Sometimes/never	180 (48.4)	148 (39.4)	
No sex past 3 months	25 (6.7)	39 (10.4)	
Tobacco use			
Daily	4 (0.8)	39 (7.9)	<0.001
Weekly	16 (3.2)	46 (9.3)	
Never	480 (96.0)	408 (82.8)	
Ever pregnant			
Yes	42 (8.4)	–	
No	458 (91.6)	–	
Circumcised			
Yes	–	300 (60.9)	
No	–	193 (39.2)	
Age at circumcision (years)			
0–10	–	30 (10.0)	
11–15	–	222 (74.0)	
16–22	–	48 (16.0)	

* Restricted to sexually experienced participants. One male and one female had missing data for ages of first and current sex partners.

† Chi-square or fisher's exact *P*-value.

Overall, females had a higher HPV prevalence than males (Table 2). In females, any HPV (i.e., detection of ≥1 HPV type) prevalence was 63.0%, and in HPV-positive females, 73.3% had multiple HPV types detected. Vaccine-type prevalence was 21.0% (HPV 16/18: 14.2%, HPV 6/11: 11.0%) and non-vaccine-type prevalence was 60.4%. In males, any HPV prevalence was 31.4%, and in HPV-positive males 40.7% had multiple HPV types detected. Vaccine-type prevalence was 9.7% (HPV 16/18: 5.3%, HPV 6/11: 5.5%) and non-vaccine-type prevalence was 28.4%. HPV prevalence was higher in sexually experienced females and males, but compared to all participants, the magnitude of differences between females and males was similar.

**Table 2.** HPV prevalences and number of HPV types detected in males and females, overall and among sexually experienced participants

HPV types detected^a^	All Participants (*N* = 993)			Sexually Experienced Participants (*N* = 748)		
	Females (*N* = 500) *n* (%)	Males (*N* = 493) *n* (%)	Chi-square *P*-value	Females (*N* = 372) *n* (%)	Males (*N* = 376) *n* (%)	Chi-square *P*-value
Any HPV	315 (63.0)	155 (31.4)	<0.001	280 (75.3)	140 (37.2)	<0.001
Quadrivalent	105 (21.0)	48 (9.7)	<0.001	99 (26.6)	43 (11.4)	<0.001
LR-HPV6/11	55 (11.0)	27 (5.5)	0.002	52 (14.0)	23 (6.1)	<0.001
HR-HPV16/18	71 (14.2)	26 (5.3)	<0.001	67 (18.0)	25 (6.7)	<0.001
Non-quadrivalent	302 (60.4)	140 (28.4)	<0.001	270 (72.6)	128 (34.0)	<0.001
Other HR-HPV	253 (50.6)	104 (21.1)	<0.001	230 (61.8)	95 (25.3)	<0.001
Other LR-HPV	217 (43.4)	72 (14.6)	<0.001	196 (52.7)	68 (18.1)	<0.001
Number of HPV types detected *(among HPV* *+* *)*			<0.001			<0.001
1	84 (26.7)	92 (59.4)		62 (22.1)	81 (57.9)	
2–4	150 (47.6)	55 (35.5)		141 (50.4)	51 (36.4)	
≥5	81 (25.7)	8 (5.2)		77 (27.5)	8 (5.7)	

HPV, human papillomavirus; LR, low risk; HR, high-risk.

Quadrivalent: HPV 6, 11, 16, 18; Quadrivalent LR: HPV 6,11; Quadrivalent HR: HPV 16, 18; Non-quadrivalent: HPV 26, 31, 33, 35, 39, 40, 42, 43, 44, 45, 51, 52, 53, 54, 56, 58, 59, 61, 66, 68, 69, 70, 73, 82; Non-quadrivalent other HR: HPV 31, 33, 35, 39, 45, 51, 52, 56, 58, 59, 66, 68; Non-quadrivalent other LR: HPV 26, 40, 42, 43, 44, 53, 54, 61, 69, 70, 73, 82.

a HPV type categories are not mutually exclusive or exhaustive; participants may be represented in more than one category due to multiple-type infections.

**Fig. 2.**
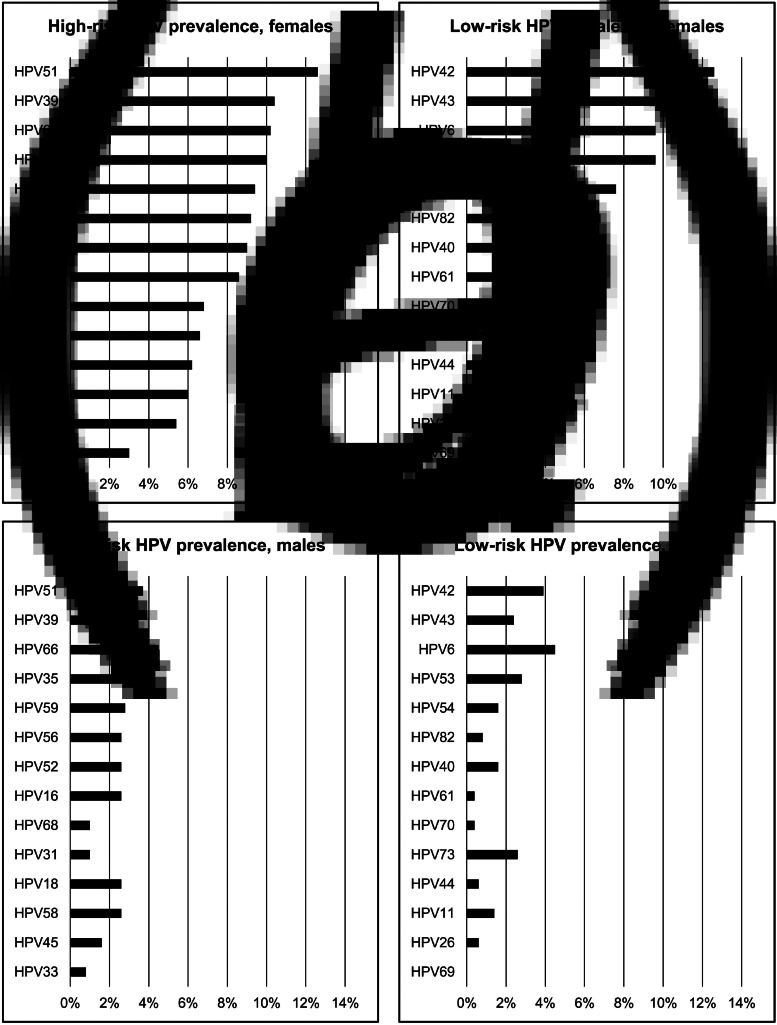
Prevalences of 14 high-risk and 14 low-risk HPV types in females and males (a) High-risk HPV prevalences, females; (b) low-risk HPV prevalences, females; (c) high-risk HPV prevalences, males; (d) low-risk HPV prevalences, males. Note: categories of high-risk HPV and low-risk HPV, and individual HPV types, are not mutually exclusive. All HPV types detected in individuals are reported; therefore, prevalences across all types sum to >100%.

The most common HR-HPV and LR-HPV types detected were similar in females and males (Fig. 2). The five most common HR-HPV types in females were HPV 51 (12.6%), HPV 39 (10.4%), HPV 66 (10.2%), HPV 35 (10.0%), HPV 59 (9.4%), and in males were HPV 66 (4.5%), HPV 51 (3.7%), HPV 35 (3.0%), HPV 59 (2.8%) and HPV 58 (2.6%). HPV 6 (4.5%) was the most common LR-HPV type in males and the fourth most common in females (9.6%).

In bivariate analyses, the prevalence of any HPV was significantly higher in females who were older, sexually experienced, had an older age of current sex partner, higher number of sex partners in the past 12 months or lifetime, sometimes or never used condoms in the past 3 months, and were daily smokers (Table 3). In males, the prevalence of any HPV was significantly higher in those who were sexually experienced, had first sex at age <18 years, had a higher number of sex partners in the past 12 months or lifetime, and sometimes or never used condoms in the past 3 months.

**Table 3.** HPV prevalences* in females and males by selected participant characteristics

	Females *N* = 500			Males *N* = 493		
	Any HPV *n* (%)	Quadrivalent HPV-type *n* (%)	Non-quadrivalent HPV-type *n* (%)	Any HPV *n* (%)	Quadrivalent HPV-type *n* (%)	Non-quadrivalent HPV-type *n* (%)
Age (years)	†	†	†		†	
18–19	147 (55.5)	40 (15.1)	139 (52.5)	62 (27.6)	15 (6.7)	57 (25.3)
20–22	168 (71.5)	65 (27.7)	163 (69.4)	93 (34.7)	33 (12.3)	83 (31.0)
Sexually experienced	†	†	†	†	†	†
No	35 (27.4)	6 (4.7)	32 (25.0)	15 (12.8)	5 (4.3)	12 (10.3)
Yes	280 (75.3)	99 (26.6)	270 (72.6)	140 (37.2)	43 (11.4)	128 (34.0)
Age of first sex (years)*				†		†
≥18	229 (74.6)	81 (26.4)	220 (71.7)	71 (32.9)	24 (11.1)	64 (29.6)
<18	51 (78.5)	18 (27.7)	50 (76.9)	69 (43.1)	19 (11.9)	64 (40.0)
Age of first sex partner (years)*						
≥20	179 (76.2)	70 (29.8)	171 (72.8)	25 (39.7)	5 (7.9)	24 (38.1)
<20	101 (74.3)	29 (21.3)	99 (72.8)	115 (36.7)	38 (12.1)	104 (33.2)
Age of current sex partner (years)*	†	†				
≥22	163 (79.5)	64 (31.2)	157 (76.6)	12 (44.4)	3 (11.1)	12 (44.4)
<22	117 (70.5)	35 (21.1)	113 (68.1)	128 (36.8)	40 (11.5)	116 (33.3)
Number of sex partners past 12 months*	†	†	†	†		†
1	159 (69.1)	48 (20.9)	152 (66.1)	47 (30.3)	15 (9.7)	42 (27.1)
2	89 (84.8)	36 (34.3)	87 (82.9)	42 (36.8)	15 (13.2)	37 (33.5)
≥3	32 (88.9)	15 (41.7)	31 (86.1)	51 (48.1)	13 (12.3)	49 (46.2)
Lifetime no. sex partners*	†	†	†	†		†
0	35 (27.3)	6 (4.7)	32 (25.0)	15 (12.8)	5 (4.3)	12 (10.3)
1	82 (64.1)	22 (17.2)	76 (59.4)	15 (22.8)	7 (11.1)	12 (19.1)
2	79 (75.2)	30 (28.6)	77 (73.3)	20 (31.3)	6 (9.4)	17 (26.6)
3–5	89 (86.4)	35 (34.0)	87 (84.5)	58 (38.9)	16 (10.7)	54 (36.2)
≥6	30 (83.3)	12 (33.3)	30 (83.3)	47 (47.0)	14 (14.0)	45 (45.0)
Condom use in the past 3 months‡	†	†	†	†	†	†
Always	111 (66.5)	36 (21.6)	110 (65.9)	59 (31.2)	16 (8.5)	57 (30.2)
Sometimes/Never	154 (85.6)	59 (32.8)	146 (81.1)	74 (50.0)	24 (16.2)	65 (43.9)
No Sex Past 3 Months	15 (60.0)	4 (16.0)	14 (56.0)	7 (18.0)	3 (7.7)	6 (15.4)
Tobacco use	†	†	†		†	
Never	302 (62.9)	99 (20.6)	289 (60.2)	126 (30.9)	40 (9.8)	114 (27.9)
Daily	4 (100.0)	3 (75.0)	4 (100.0)	12 (30.8)	2 (5.1)	12 (30.8)
Weekly	9 (56.3)	3 (18.8)	9 (56.3)	17 (37.0)	6 (13.0)	14 (30.4)
Circumcised						
Yes	–	–	–	88 (29.3)	23 (7.7)	78 (26.0)
No	–	–	–	67 (34.7)	25 (13.0)	62 (32.1)
Age at circumcision (years)					†	
0–10	–	–	–	11 (36.7)	3 (10.0)	10 (33.3)
11–15	–	–	–	60 (27.0)	12 (5.4)	55 (24.8)
16–22	–	–	–	17 (35.4)	8 (16.7)	13 (27.1)

HPV, human papillomavirus; Quadrivalent HPV: HPV 6, 11, 16 and 18; Non-quadrivalent HPV: HPV 26, 31, 33, 35, 39, 40, 42, 43, 44, 45, 51, 52, 53, 54, 56, 58, 59, 61, 66, 68, 69, 70, 73, 82.

* Quadrivalent and non-quadrivalent HPV-type categories are not mutually exclusive.

† Chi-square or fisher's exact test *P*-value <0.05.

‡ Among sexually experienced participants only.

In multivariable analyses limited to sexually experienced participants, the number of sex partners in the past 12 months and condom use remained significantly associated with any HPV prevalence in females and males (Table 4). Compared to those reporting 0–1 sex partner in the past 12 months, the prevalence of any HPV in those reporting 2 or more sex partners was 21% higher in females (aPR 1.21, 95% CI 1.10–1.34) and 33.0% higher in males (aPR 1.33, 95% CI 1.01–1.76). Compared to participants who reported only occasionally or never using condoms in the past 3 months, those reporting always using condoms had a 20.0% lower prevalence of any HPV in females (aPR 0.80, 95% CI 0.71–0.90) and a 36.0% lower prevalence in males (aPR 0.64, 95% CI 0.49–0.83). Prevalence of any HPV was 29.3% in males who were circumcised compared to 34.7% in males who were not circumcised (aPR 0.82, 95% CI 0.64–1.05).

**Table 4.** Unadjusted and adjusted prevalence ratios for the prevalence of any HPV by participant characteristics in sexually experienced females and males

	PR (95% CI)	aPR (95% CI)
Females		
Number of sex partners past 12 months		
0–1	Ref	Ref
≥2	1.25 (1.12–1.39)	1.21 (1.10–1.34)
Condom use in past 3 months		
Sometimes/never	Ref	Ref
Always	0.78 (0.69–0.88)	0.80 (0.71–0.90)
No sex in past 3 months	0.70 (0.51–0.97)	0.71 (0.52–0.98)
Males		
Number of sex partners past 12 months		
0–1	Ref	Ref
≥2	1.40 (1.06–1.86)	1.33 (1.01–1.76)
Condom use in past 3 months		
Sometimes/never	Ref	Ref
Always	0.62 (0.48–0.81)	0.64 (0.49–0.83)
No sex in past 3 months	0.36 (0.18–0.72)	0.39 (0.19–0.77)
Circumcised		
No	Ref	Ref
Yes	0.82 (0.63–1.07)	0.82 (0.64–1.05)

Any HPV, any of the 28 HPV types detected by the Anyplex II HPV28 assay; PR, prevalence ratio; aPR, adjusted prevalence ratio; CI, confidence interval. Multivariable models were developed using backward selection (*P* < 0.3 to enter, *P* < 0.15 to stay); variables considered in models included age, age of first sexual experience, age of current and first sex partners, number of sex partners past 12 months, condom use in past 3 months, tobacco use, and among males, circumcision.

## Discussion

This cross-sectional study is the first to characterise HPV prevalence in college-attending, vaccine-naive young adult females and males aged 18–22 years in Gaborone, Botswana. Importantly, we report the prevalence of HPV types targeted by the quadrivalent vaccine, which can be used as a baseline for monitoring future HPV vaccine impact in females and herd protection from the female vaccination programme in males.

The prevalence of any HPV type in females in this report (63.0%) was similar or higher than in other studies of HPV prevalence in females of comparable age in Africa. In a meta-analysis of HPV types detected in African women aged 15–24 years who had normal cytology, the prevalence of any HPV was 48.2% (95% CI 14.7–81.6) [14]. In more recent studies in HIV-negative females, the prevalence of any HPV was very similar to this report, 61.0% in South Africa [15] and 63.6% in Mozambique [16]. However, the prevalence was lower in a report of university students in Mozambique (28.6%) [17] and in females in Rwanda (42.4%) [18]. The differences in the prevalence across studies may be due to differences in sexual behaviour, specimen collection, inclusion criteria (e.g., excluding abnormal cytology), or differences in HPV assays used, including assay sensitivity and the number of types they detect. Of note, HPV assays in these studies were the same as the one used in this report or were assays that could detect a greater number of HPV types.

Few prior studies have reported on HPV prevalence in young adult males in sub-Saharan Africa [19], and even fewer have reported on HPV prevalence in males and females from the same population [15, 16]. In our report, the prevalence of any HPV type in young adult males (37.2%) was lower than that observed in young adult males in Kenya (50.0%) [20], South Africa (77.0%) [15] and Uganda (42.8%) [21], and higher than in Tanzania (20.5%) [22], and Mozambique (10.2%) [16]. The reasons for the differences in prevalence may be due to sampling techniques (self versus provider), dry or wetted swab, sexual risk behaviour, and anatomical site(s) sampled. For example, lower HPV prevalence was observed in a Mozambique study where specimens were taken from the urethra, which is known to harbour less HPV than other penile sites [16, 19]. Higher HPV prevalence was observed in a Kenya study where the participants had higher risk sexual behaviours [20]. The few studies comparing HPV prevalence in males and females in Africa show mixed results [15, 16]. However, a study in the United States found that HPV prevalence was higher in females than males in 14–19 and 20–24-year-olds, and higher in males than females in older age groups [23]. Although the males were more sexually active in this study, their lower HPV prevalence could be explained, in part, by sampling error. Unlike sampling a moist surface such as the cervix, reliable sampling of the dry, keratinised surface of the penis to obtain adequate numbers of cells for HPV testing is more difficult. The lower HPV prevalence in males may also be due to the age of sex partners. We found that the men have much younger first and current sex partners than the women. The age of the sex partner influences the likelihood that the partner is infected; having older sex partners is a risk factor for sexually acquired infections [24].

Similar to most studies conducted in sub-Saharan Africa, the majority of HPV-positive participants in this study had multiple HPV types detected (73.3% in females and 41.9% in males). A study in South Africa reported a multiple-type prevalence of 31.7% in females and 48.6% in males aged 18–25 years [15], and a study in Mozambique reported a multiple-type prevalence of 58.7% in females and 27.8% in males aged 18–24 years [16]. Infection with multiple HPV types has been associated with higher HPV persistence [25] and a greater likelihood of progression to pre-cancer and cancer [26].

Due to the recent initiation of most HPV vaccination programmes in Africa, Rwanda is the only country which has reported HPV vaccine impact [27]. A few other studies, however, have reported vaccine-type HPV prevalence [28]. A study in 16–24-year-old females in South Africa reported 20.0% quadrivalent vaccine-type prevalence, similar to our report. In another study in persons aged 18–24 years in Mozambique, the prevalence of quadrivalent vaccine types was 26.2% in females, but only 2.8% in males [16]. A study in South Africa reported lower quadrivalent vaccine-type prevalence, 12.0% and 13.0%, in HIV-negative females and males, respectively, than we report. However, that study included persons aged 18–66 years and did not report age-specific data [15].

Using multivariable modelling to understand associations between participant characteristics and prevalence, we identified the number of sex partners in the past 12 months and condom use as the characteristics most strongly associated with the prevalence of any HPV in females and males. The number of sex partners is a well-known risk factor for HPV infection and has also been identified in other studies in university students in Africa [17]. Consistent condom use also has been shown to reduce HPV infections, as observed in this study [29, 30]. The protective effect of circumcision did not reach statistical significance in our study, possibly due to low statistical power. A meta-analysis of data through 2015, including pooled data from 19 observational studies and four randomised controlled trials, found that circumcised men have a reduced odds of genital HPV prevalence [31]. Variability in sampling methods, HPV DNA detection assays, and specimen collection sites could contribute to the variation in effects.

A major strength of this study is the concurrent sampling of females and males and the HPV typing with the same assay in the same laboratory. Importantly, although this study was conducted after the start of the national HPV immunisation programme, we enrolled females who were not eligible for vaccination and, therefore, were able to obtain an unvaccinated baseline. Although it could not be assessed in this study, depending on ages of sex partners, indirect effects from vaccinated younger females could have impacted the baseline HPV prevalence in unvaccinated females and males aged 18–22 years in this study, but we expect any such indirect effects to be small and to have minimal impact on a future vaccine impact evaluation. In 2024, the first vaccinated girls will reach the age group enrolled in this study and another cross-sectional study will allow evaluation of the impact of the vaccination programme. While a previous evaluation in Rwanda reported impact in the first country in Africa to introduce HPV vaccination, that country started their programme with a 3-dose schedule [24]. The future evaluation in Botswana will be able to evaluate the impact of a 2-dose HPV vaccination programme in Africa, as well as herd protection in males.

The study had some limitations. We used convenience sampling at one university; therefore, the HPV prevalence estimates may not be fully generalisable to the target population of males and females aged 18–22 years but repeating the same recruitment methods in 4–5 years for a follow-up study to assess vaccine impact should yield a comparable population. Although the study enrolled participants from one site, this study still included a diverse population. Students come from across the country to attend this one major university in Botswana.

In conclusion, the findings from this study indicate a high prevalence of HPV, including HPV types targeted by the quadrivalent vaccine, in both females and males in Botswana. The national HPV vaccination programme in Botswana, which achieved vaccination coverage as high as 90.0% (personal communication from Botswana Ministry of Health), is thus expected to have a substantial impact on reducing HPV infection and HPV-associated cancers. The data from this study provide a baseline HPV prevalence in Botswana that will facilitate evaluation of HPV vaccine impact in the future, including direct impact in young women and potential herd protection in young men.

## Data

Anonymised data from this study are available on request from the corresponding author.

## References

[1] Forman D (2012) Global burden of human papillomavirus and related diseases. Vaccine 30, F12–F23. 23199955 10.1016/j.vaccine.2012.07.055

[2] de Martel C (2017) Worldwide burden of cancer attributable to HPV by site, country, and HPV type. International Journal of Cancer 141, 664–670. 28369882 10.1002/ijc.30716 PMC5520228

[3] Sung H (2021) Global cancer statistics 2020: GLOBOCAN estimates of incidence and mortality worldwide for 36 cancers in 185 countries. CA: a cancer journal for clinicians 71, 209–249. 33538338 10.3322/caac.21660

[4] Markowitz LE (2012) Human papillomavirus vaccine introduction--the first five years. Vaccine 30, F139–F148. 23199957 10.1016/j.vaccine.2012.05.039

[5] Schiller JT, Castellsagué X and Garland SM (2012) A review of clinical trials of human papillomavirus prophylactic vaccines. Vaccine 30,5, F123–F138. 23199956 10.1016/j.vaccine.2012.04.108 PMC4636904

[6] Joura EA, (2015) A 9-valent HPV vaccine against infection and intraepithelial neoplasia in women. The New England Journal of Medicine 372, 711–723. 25693011 10.1056/NEJMoa1405044

[7] HPV Vaccines: Vaccinating Your Preteen or Teen. Available at https://www.cdc.gov/hpv/parents/vaccine.html. (Accessed May 2021).

[8] Ramogola-Masire D. HPV Vaccine for cervical cancer prevention in Botswana. Available at http://www.commonwealthhealth.org/wp-content/uploads/2014/05/7-HPV-botswana-Masire.pdf (Accessed June 1 2021).

[9] Raesima MM (2015) Human papillomavirus vaccination coverage among school girls in a demonstration project – Botswana, 2013. MMWR. Morbidity and Mortality Weekly Report 64, 1147–1149. 26468997 10.15585/mmwr.mm6440a5

[10] Binagwaho A (2012) Achieving high coverage in Rwanda's national human papillomavirus vaccination programme. Bulletin of the World Health Organization 90, 623–628. 22893746 10.2471/BLT.11.097253 PMC3417784

[11] Drolet M (2015) Population-level impact and herd effects following the introduction of human papillomavirus vaccination programmes: a systematic review and meta-analysis. Lancet Infectious Diseases 15, 565–580. 25744474 10.1016/S1473-3099(14)71073-4 PMC5144106

[12] Drolet M, (2019) Population-level impact and herd effects following the introduction of human papillomavirus vaccination programmes: updated systematic review and meta-analysis. Lancet 394, 497–509. 31255301 10.1016/S0140-6736(19)30298-3 PMC7316527

[13] McClung N (2022) HPV Prevalence among young adult women living with and without HIV in Botswana for future HPV vaccine impact monitoring. BMC Infectious Diseases 22, 176. 35193517 10.1186/s12879-022-07130-x PMC8862300

[14] Ogembo RK (2015) Prevalence of human papillomavirus genotypes among African women with normal cervical cytology and neoplasia: a systematic review and meta-analysis. PloS One 10, e0122488. 25875167 10.1371/journal.pone.0122488 PMC4396854

[15] Mbulawa ZZ, Coetzee D and Williamson AL (2015) Human papillomavirus prevalence in South African women and men according to age and human immunodeficiency virus status. BMC Infectious Diseases 15, 459. 26502723 10.1186/s12879-015-1181-8 PMC4624185

[16] Edna Omar V (2017) Human papillomavirus prevalence and genotype distribution among young women and men in Maputo city, Mozambique. BMJ Open 7, e015653. 10.1136/bmjopen-2016-015653 PMC5722086 28716790

[17] Bule YP (2020) Human papillomavirus prevalence and distribution in self-collected samples from female university students in Maputo. International Journal of Gynaecology and Obstetrics: The Official Organ of the International Federation of Gynaecology and Obstetrics 149, 237–246. 10.1002/ijgo.13126 32086940

[18] Ngabo F (2016) Human papillomavirus infection in Rwanda at the moment of implementation of a national HPV vaccination programme. BMC Infectious Diseases 16, 225. 27221238 10.1186/s12879-016-1539-6 PMC4877733

[19] Olesen TB (2014) Human papillomavirus prevalence among men in sub-Saharan Africa: a systematic review and meta-analysis. Sexually Transmitted Infections 90, 455–462. 24812407 10.1136/sextrans-2013-051456

[20] Smith JS (2007) Human papillomavirus detection by penile site in young men from Kenya. Sexually Transmitted Diseases 34, 928–934. 17621251 10.1097/OLQ.0b013e318065b8ef PMC2519883

[21] Tobian AA (2013) High-risk human papillomavirus prevalence is associated with HIV infection among heterosexual men in rakai, Uganda. Sexually Transmitted Infections 89, 122–127. 22628661 10.1136/sextrans-2012-050524 PMC3640492

[22] Olesen TB (2013) Prevalence and type distribution of human papillomavirus among 1813 men in Tanzania and the relationship to HIV status. Sexually Transmitted Diseases 40, 592–598. 23965778 10.1097/OLQ.0b013e31828fcf57

[23] Lewis RM (2018) Prevalence of genital human papillomavirus among sexually experienced males and females aged 14–59 years, United States, 2013–2014. The Journal of Infectious Diseases 217, 869–877. 29294016 10.1093/infdis/jix655 PMC5991084

[24] Topazian HM (2020) Variations in HIV risk by young women's age and partner age disparity in rural South Africa (HPTN 068). Journal of Acquired Immune Deficiency Syndromes (1999)83, 350–356. 31904708 10.1097/QAI.0000000000002270 PMC7722780

[25] Ho GY (1998) Natural history of cervicovaginal papillomavirus infection in young women. The New England Journal of Medicine 338, 423–428. 9459645 10.1056/NEJM199802123380703

[26] Trottier H (2006) Human papillomavirus infections with multiple types and risk of cervical neoplasia. Cancer Epidemiology, Biomarkers & Prevention 15, 1274–1280. 10.1158/1055-9965.EPI-06-0129 16835323

[27] Baussano I (2021) Impact of human papillomavirus vaccination, Rwanda, and Bhutan. Emerging Infectious Diseases 27, 1–9. 10.3201/eid2701.191364 PMC7774553 33350922

[28] Giuliano AR (2015) High HIV, HPV, and STI prevalence among young Western Cape, South African women: EVRI HIV prevention preparedness trial. Journal of Acquired Immune Deficiency Syndromes 68, 227–235. 25415290 10.1097/QAI.0000000000000425 PMC4378717

[29] Rodríguez-Álvarez MI (2018) Prevalence and risk factors of human papillomavirus in male patients: a systematic review and meta-analysis. International Journal of Environmental Research and Public Health 15, 2210. 10.3390/ijerph15102210 PMC6210641 30309014

[30] Winer RL (2006) Condom use and the risk of genital human papillomavirus infection in young women. The New England Journal of Medicine 354, 2645–2654. 16790697 10.1056/NEJMoa053284

[31] Zhu YP (2017) Relationship between circumcision and human papillomavirus infection: a systematic review and meta-analysis. Asian Journal of Andrology 19, 125–131. 26975489 10.4103/1008-682X.175092 PMC5227661

